# Phenotypic and functional alterations of peritoneal macrophages in lupus-prone mice

**DOI:** 10.1007/s11033-022-07252-0

**Published:** 2022-02-24

**Authors:** Gabriela Tejon, Nicolás Valdivieso, Felipe Flores-Santibañez, Verónica Barra-Valdebenito, Víctor Martínez, Mario Rosemblatt, Daniela Sauma, María Rosa Bono

**Affiliations:** 1 Departamento de Biología, Facultad de Ciencias, Universidad de Chile, Santiago, Chile; 2 Facultad de Medicina y Ciencia, Universidad San Sebastián, Santiago, Chile; 3 FAVET-INBIOGEN, Facultad de Ciencias Veterinarias, Universidad de Chile, Santiago, Chile; 4 Centro Ciencia & Vida, Santiago, Chile

**Keywords:** Autoimmunity, Macrophage, Systemic lupus erythematosus (SLE), Peritoneal cavity

## Abstract

**Background:**

Several studies have demonstrated the contribution of innate immune cells, including macrophages, in promoting systemic lupus erythematosus (SLE). Macrophages, one of the most abundant cell populations in the peritoneal cavity, are considered multifunctional cells with phenotypic plasticity. However, the functional properties of peritoneal macrophages in steady-state and during the progression of SLE remain poorly defined.

**Methods and results:**

Using the [NZB × NZW]F1 (BWF1) murine model of SLE, we analyzed the phenotype and function of peritoneal macrophages during the disease’s onset. We found a higher frequency of peritoneal macrophages and B1a cells in BWF1-diseased mice than age-matched controls. Additionally, macrophages from diseased animals expressed lower levels of CD206, MHC-II, and Sirpα. RNAseq analysis identified 286 differentially expressed genes in peritoneal macrophages from diseased-BWF1 mice compared to control mice. Functional experiments demonstrate that peritoneal macrophages from diseased-BWF1 mice secrete higher levels of pro-inflammatory cytokines when activated with TLR7 and TLR9 agonists, and they were less efficient in suppressing the activation and proliferation of peritoneal LPS-activated B cells. These data demonstrate that peritoneal macrophages from BWF1-diseased mice present phenotypic and functional alterations shifting to a more pro-inflammatory state.

**Conclusions:**

The increase of macrophages with an altered phenotype and function together with the accumulation of B1a cells in the peritoneal cavity of diseased-BWF1 mice may promote the progression of the disease. Advancing awareness of the role and phenotype of peritoneal macrophages in SLE may contribute to a better understanding of these types of diseases and the development of novel therapies.

**Supplementary Information:**

The online version contains supplementary material available at 10.1007/s11033-022-07252-0.

## Introduction

Systemic lupus erythematosus (SLE) is a multifactorial systemic autoimmune disease that affects multiple organs and tissues. SLE is characterized by the overactivation of T and B lymphocytes, the subsequent accumulation of autoantibodies against nuclear and cytoplasmic antigens, and overexpression of pro-inflammatory cytokines [1, 2]. These pathogenic autoantibodies deposit as immune complexes in diverse tissues and organs, generating inflammation and damage [3]. Although defects in adaptive immunity are an important cause of SLE, studies in patients and animal models suggest that innate immune cells, including macrophages, are involved in the pathogenesis of this disease [4, 5].

Recent studies that focus on the role of macrophages in SLE show contradicting results, where the pharmacological depletion of this population of cells has shown to either promote or delay the disease in murine models of SLE [6, 7]. Moreover, several groups have observed profound alterations in the cytokine production profile in macrophages from MRL^+/+^ and BWF1 mice, two murine models of SLE [8]. In addition, it has been reported that macrophage-derived IL-6 has a prominent role in promoting anti-DNA autoantibodies in lupus-prone BWF1 mice [9].

Some studies have shown that macrophages and B cells are the main populations in the peritoneal cavity, where the B‑1a subpopulation is the most abundant within peritoneal B cells [10]. B-1a cells produce autoantibodies and thus have been linked to the development of autoimmune diseases. On the other hand, peritoneal macrophages (PMs) are critical players in controlling infectious and inflammatory diseases [11, 12]. These cells also regulate peritoneal cavity homeostasis by the phagocytosis of apoptotic and necrotic bodies [13]. Thus, alterations in their function have been linked to autoimmunity as well [14, 15]. Interestingly, it has been demonstrated that peritoneal macrophages modulate peritoneal B cell proliferation and migration into the peritoneal cavity [16], suggesting complex crosstalk between these two populations.

Few studies have analyzed the role of peritoneal cavity macrophages in SLE. In this line, one study has demonstrated functional alterations and abnormal uptake of apoptotic bodies in PMs from MRL/Mp and NZB/W mice [17]. Additionally, peritoneal macrophages from B6.MRL-Fas^*lpr*^ mice have a decreased expression of CD206, one of the well-accepted markers for M2 macrophages, and decreased phagocytic activity when exposed in vitro to FluorSpheres [18]. However, the functional properties of peritoneal macrophages in steady-state and during the progression of this autoimmune disease remain poorly defined.

Given the well-known interaction between peritoneal macrophages and autoantibody-producing B1 lymphocytes [19], we decided to study peritoneal macrophages in BWF1 mice, a murine model of SLE. We also focused on the interaction between peritoneal macrophages and B cells. We found a higher frequency of PMs and B1a cells in BWF1-diseased mice compared to control mice. Also, we demonstrate that PMs from BWF1-diseased mice have lower expression of mannose receptor (CD206), MHC-II, and Sirpα, suggesting a shifting from the M2 phenotype. RNAseq assays confirmed that 286 genes are differentially expressed in PMs from diseased mice. Additionally, we analyzed the response of PMs to different TLR agonists and observed that PMs from diseased-BWF1 mice secrete higher levels of different cytokines than age-matched control. Finally, we observed that peritoneal macrophages from diseased mice were less efficient in suppressing the activation and proliferation of peritoneal LPS-activated B cells. Altogether, these data demonstrate that PMs have an altered frequency, and acquire a more pro-inflammatory phenotype and function during lupus-like disease. Thus, modulation of PMs may be an attractive therapy for SLE.

## Materials and methods

### Mice

Female lupus-prone [NZBxNZW]F1 (BWF1) mice were purchased from The Jackson Laboratory (Bar Harbor, ME, USA). All mice used in this study were housed following the institutional regulations in the animal facility of Fundación Ciencia & Vida. BWF1 female mice (H2^z x d^ haplotype) between 3 and 5-months-old were used to describe a pre-lupic status that still does not develop autoimmune disease. Diseased-BWF1 mice had high antibody titers against double-stranded DNA (dsDNA) and severe proteinuria (i.e., ≥ 500 mg/dl protein) on three consecutive measurements. Since there are gender differences in the development of this disease, we used female non-autoimmune mice as controls with the same H2 haplotype as BWF1 mice. Thus, age-matched [NZW × BALB/c]F1 female mice were used as non-autoimmune controls. Proteinuria was measured monthly during the first 6 months of age by a standard semi-quantitative test using a Combur Test N (Roche Diagnostics, Germany). After 6 months of age, proteinuria was measured every week to detect premature lupus. Autoantibodies against dsDNA were measured in serum samples by a standard ELISA.

### Isolation of peritoneal cells

Peritoneal cavity (PerC) cells were isolated from mice as previously described [20]. Briefly, mice were euthanized, the inner peritoneal skin was exposed, and 10 mL of sterile PBS was injected into this cavity. Following a gentle massage of the peritoneum, the peritoneal fluid was collected and centrifuged at 600 g for 7 min. Cells were resuspended in Red Blood Cell (RBC) lysis buffer (Biolegend) and ice-incubated for 5 min. After washing the cells, they were resuspended in RPMI 1640 with 10% fetal calf serum (FCS) for further analysis.

Peritoneal macrophages and B cells were further purified by cell sorting based on CD19 and F4/80 staining. Macrophages were defined as F4/80 + /CD19-/CD11b high whereas B cells were defined as CD19 + /F4/80-. The purity of macrophages and B cells was always ≥ 95 and 93% respectively.

### Flow cytometry

Surface staining was performed in ice-cold PBS with 2% FCS for 15 min in the presence of FcγRII/III blocking antibody (CD16/32). The cells were surface-stained with the relevant antibodies in PBS + 2% FBS for an additional 20 min at 4 °C.

The following fluorochrome-conjugated monoclonal antibodies were used for cell phenotyping: B220 APC (clone RA3-6B2), CD11b PE (clone M1-70), CD138 PE (clone 281-2), CD19 FITC (clone 6D5), CD19 BV421 (clone C068c2), CD206 PE-Cy7 (clone C068c2), CD4 PE (clone RM4-5), CD5 PE-Cy7 (clone 53–7.3), CD8a Ly-2 APC/Fire 750 (clone 53–6.7), F4/80 APC-Cy7 (clone BM8), F4/80 APC (clone BM9), IA-d Alexa Fluor 647 (clone 39–10-8), SIRPα APC (clone 15–414) from Biolegend and CD3 eFlour 660 (clone 17A2). Viability dye eFluor 780 reagent (eBioscience, USA) or propidium iodide were used for live/dead cell discrimination.

Flow cytometry was conducted on a FACSCanto II flow cytometer or FACSAria III Cell Sorter (BD Biosciences), and data analysis was performed using the FlowJo software (Tree Star, Inc., Ashland, OR, USA).

### In vivo engulfment assay

Syngeneic thymocytes were cultured overnight with 0.1 mM dexamethasone to induce apoptosis. After that, apoptotic thymocytes (annexin V + cells) were stained with CellTrace Violet (Invitrogen) according to the manufacturer’s instructions. 5 × 10^6^ labeled apoptotic thymocytes were injected i.p. to diseased-BWF1 and age-matched control mice. After 45 min, peritoneal cells were harvested and labeled with F4/80, CD19, CD4, and CD8 antibodies for flow cytometry analysis.

### Macrophage in vitro stimulation

PMs from diseased-BWF1 and age-matched control mice were isolated by cell sorting (F4/80 + , CD19-, CD11b high) with a purity of ≥ 95%. After cell-sorting, cells were washed with Penicillin 300 U/mL and Streptomycin 0.3 mg/mL media to avoid any contamination of the cultures and resuspended in culture media (RPMI 1640 with 10% FCS + 2-Mercaptoethanol 0.055 μM + Fungizone 0.5 μg/mL). Macrophages were activated with 1 μg/mL LPS from *E. coli* O111:B4 (Sigma-Aldrich), 1 μg/mL R848 Resiquimod or 10 μg/mL CpG ODN 1585 (Invivogen) overnight at 37 °C, 5% CO_2_. Following activation, the supernatants were harvested and analyzed using the CBA Mouse Inflammation Kit (BD Bioscience) according to the manufacturer’s instructions.

### Macrophage and B cell co-cultures

Cell-sorted PMs from diseased-BWF1 and aged-matched control mice were cocultured with sorted aged-matched control peritoneal B cells (CD19 +) in the presence of diverse stimuli. Sorted total B cells were stained with 5 μM CFSE (Molecular Probes, Eugene, OR, USA) according to the manufacturer’s instructions and resuspended at 2 × 10^6^ cells/mL. The coculture was performed with 20,000 macrophages and 100,000 B cells per well in a P96 plate. The stimuli used were 2 ng/mL LPS from *E. coli* O111:B4 (Sigma-Aldrich), 1 μg/mL CpG ODN 1826 (Invivogen) or 2 μg/mL α-CD40 + 2 ng/mL IL-4 (Biolegend). The cells were cultured for four days and then stained for flow cytometry with CD19, CD138, and viability dye to exclude dead cells. Proliferation was analyzed by CFSE dilution.

### RNAseq

RNA from cell-sorted Peritoneal Macrophages (CD11b + F4/80 + CD19-) was extracted using TRIzol reagent (Life Technologies) according to the manufacturer's instructions. Purified RNA samples were quantified using Qubit RNA HS Assay (Life Technologies, Thermo Fisher Scientific) and integrity was measured using the High Sensitivity RNA Analysis Kit (Advanced Analytical Technologies). Sequencing libraries were prepared using the KAPA Stranded mRNA-Seq kit according to the manufacturer's protocol (Illumina). The length of the libraries was determined by capillary electrophoresis using the Standard Sensitivity NGS Fragment Analysis Kit (Advanced Analytical Technologies). Libraries were quantified using the KAPA Library Quantification Kit (Kappa Biosystem) using the Eco PCR system (Illumina), following the manufacturer's protocol. Libraries were sequenced on a Miseq platform (Illumina) using a v3 150 kit with 2 × 75 bp paired-end. The subsequent analysis was performed in R using the Deseq2 package to normalize the data. Differentially expressed genes were identified using an adjusted p-value cut-off of 0.05 and a fold change of 1.5. The data discussed in this publication have been deposited in NCBI’s Gene Expression Omnibus and are accessible through GEO Series accession number GSE167108.

### Statistical analysis

Statistical analysis was performed with the GraphPad Prism program V8 (GraphPad Software, San Diego, CA, USA). The data were compared using a Student’s t-test after verification of normal distribution. Mann Whitney test was used for the data that did not adjust to a normal distribution. P-values < 0.05 were considered significant.

## Results

### The peritoneal cavity of diseased-BWF1 mice harbors a higher frequency of peritoneal macrophages

**Fig. 1:**
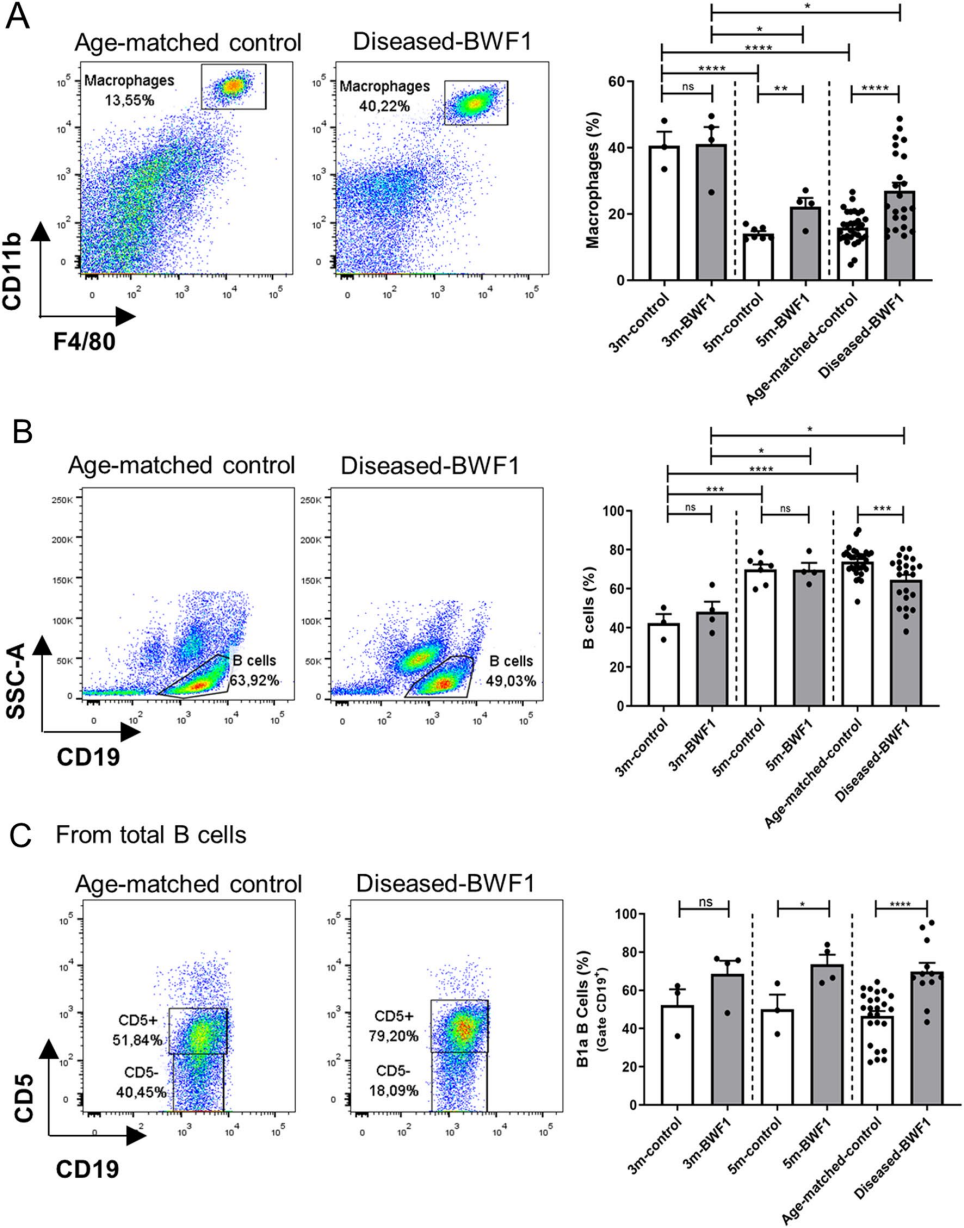
The peritoneal cavity of diseased-BWF1 mice harbors a higher frequency of peritoneal macrophages. **A** Representative FACS analysis (left) and frequency (right) of peritoneal macrophages (F4/80 + CD11b +) from diseased-BWF1 and age-matched-control mice (gated on live cells). **B** Representative pseudo-color plot (left) and frequency (right) of total peritoneal B cells (CD19 +) in BWF1 and age-matched control mice (gated on live cells). **C** Representative pseudo-color plot (left) and frequency (right) of B1a cells (CD5 + gate) in total B cells from peritoneal cavity of BWF1 and age-matched control mice. Each dot represents one mouse (n = 3–31 mice per group). Student's t-test was used in all comparations except in A when comparing the frequency of diseased-BWF1 macrophages with age-matched control, 3 m-BWF1 or 5 m-BWF1, where Mann–Whitney was used. p > 0.0 5; *p < 0.05; **p < 0.01; ***p < 0.001; ****p < 0.0001

The peritoneal cavity is home to a complex mixture of immune cells with essential functions in monitoring visceral organs and maintaining tissue homeostasis, such as peritoneal macrophages (PMs) and B cells [21]. Considering this and the possible implication of PMs in autoimmune diseases, we first analyzed the dynamics of peritoneal cell populations during SLE development. We compared cells obtained from the peritoneal exudate of BWF1 mice at different stages preceding (3 and 5 months old) and after the onset of the disease (9 months old on average) with cells from age-matched controls (NZWxBALB/c) F1 mice. In BWF1 mice we found large peritoneal macrophages defined as CD11b + and F4/80 + cells. We observed a decrease in PMs frequency with age in both BWF1 and control mice (Fig. 1A). However, at 5 months, there is a significantly higher percentage of macrophages in the peritoneal cavity of BWF1 mice than 5 m-control mice, and this difference increases in diseased-BWF1 mice, which have almost a two-fold increase in the frequency of PMs compared to age-matched control mice (27 ± 2.4% vs. 15 ± 1%). There is also an increase in macrophages absolute numbers in this condition (Control 2.6 ± 0.9 × 10^6^ and diseased-mice 4.0 ± 1.7 × 10^6^ cells).

On the other hand, when we evaluated B cells in the peritoneal cavity, we found a significant decrease in this population only in diseased-BWF1 mice (64.6 ± 2.5% vs. 73.9 ± 1.3%) compared to age-matched control mice (Fig. 1B). A relevant B cell population in autoimmune diseases is B1a cells (CD5^+^), responsible for the production of natural antibodies. As previously reported, we observed that most of B cells found in the peritoneal cavities of diseased and control mice are B1a cells (Fig. 1C) [10]. Interestingly, we observed a higher frequency of B1a cells in 5-month-old BWF1 and diseased-BWF1 mice compared to age-matched control mice.

Altogether this data demonstrates a dysregulation in the proportion of macrophages and B cells in the peritoneal cavity during the disease’s onset.

### Peritoneal macrophages from diseased-BWF1 mice are phenotypically different compared to control mice

**Fig. 2:**
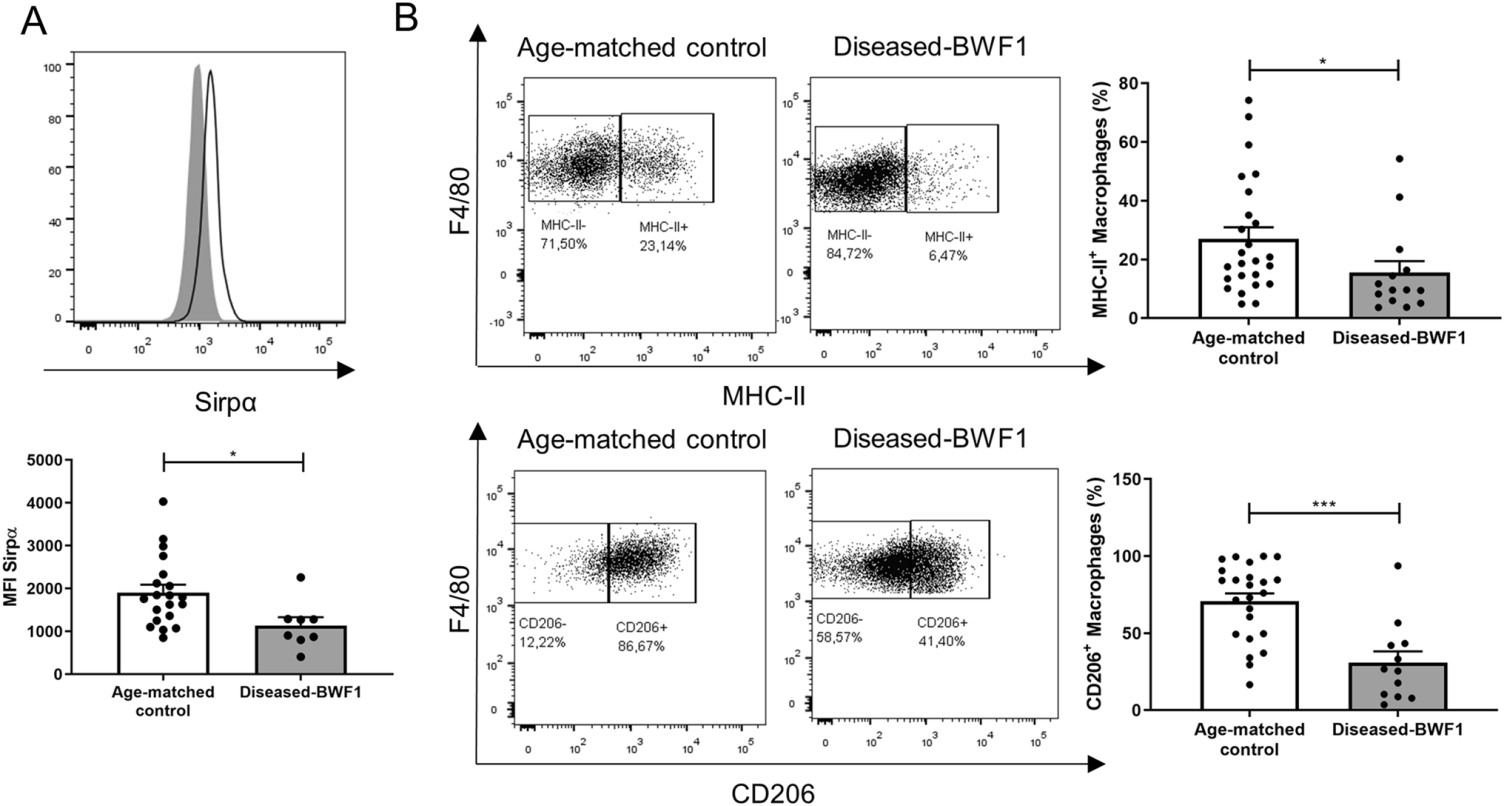
Peritoneal macrophages from diseased-BWF1 mice are phenotypically different to control mice. **A** Representative histogram and mean fluorescence intensity (MFI) of Sirpα in peritoneal macrophages (gate F4/80 + CD11b +) from diseased-BWF1 (shaded histogram) and age-matched-control mice (black line histogram). **B** Representative FACS plots depicting MHC-II and CD206 expression (left) and frequency (right) of MHC-II + and CD206 + peritoneal macrophages from diseased-BWF1 and age-matched-control mice. Each dot represents one mouse (n = 14–24 mice per group). Statistical analysis: In A Student’s t-test, in B Mann–Whitney test. *p < 0.05; ***p < 0.001

In parallel, we analyzed by flow cytometry the expression of different functional markers in PMs. We found significant differences in some proteins associated with important functions displayed by macrophages as Sirpα, MHC-II, and the M2 marker CD206 (Fig. 2). In diseased-BWF1 mice, we observed a lower frequency of MHC-II + PMs and they presented a lower mean fluorescence intensity for Sirpα (Fig. 2A and B) compared with age-matched control mice. We also found that the percentage of PMs that express CD206 is lower in diseased-BWF1 mice (30.8 ± 7.4%) when compared with PMs from age-matched control mice (70.7 ± 5.1%). These results demonstrate that macrophages residing in the peritoneal cavity present alteration in their phenotype in BWF1-diseased mice.

### Peritoneal macrophages from diseased-BWF1 show no alterations in the uptake of apoptotic cells in vivo

The engulfment of apoptotic cells and cellular debris constitutes a primary function of macrophages, and its alteration has been related to the onset of autoimmune diseases [22]. To compare the uptake capacity of apoptotic cells between PMs from diseased-BWF1 mice and age-matched control mice, we performed an in vivo assay. We adoptively transferred by intraperitoneal injection (i.p.), fluorescent-labeled apoptotic thymocytes (CellTrace Violet +) into diseased and control mice. After 45 min, we analyzed the percentage of apoptotic cells engulfed by PMs. For this, cells recovered from the peritoneal cavity were labeled with F4/80, CD19, CD4, and CD8 antibodies. Using this strategy, cell-trace violet labeled (CTV +) thymocytes that have been internalized by macrophages will not stain with anti-CD4 and CD8 antibodies (as they will be internalized and CD4/CD8 will not be available for cell surface staining) but will fall within an F4/80 + gate. On the other hand, CTV + thymocytes that only bind macrophages will be found in the F4/80 + gate and will also stain with CD4 and CD8 antibodies. Finally, free CTV + thymocytes will stain with CD4 and CD8 antibodies, but will remain F4/80 negative.

In Supplementary Fig. 1A, we show representative plots of the thymocytes that have been internalized by PMs from diseased and control mice. As shown in the bar graphs, 40.4 ± 8.3% of the apoptotic cells were internalized by macrophages from BWF1 mice whereas 32.2 ± 6% were internalized by macrophages from control mice. In addition, in our analyses, we observed that only 3–4% of the apoptotic thymocytes were internalized by B cells (data not shown). These results show no significant differences in apoptotic cell clearance by peritoneal macrophages in BWF1 versus control mice.

On the other hand, when we analyzed the percentage of macrophages that have internalized apoptotic cells (Supplementary Fig. 1B), we observed no significant differences between BWF1 and control macrophages (43.7 ± 2% of BWF1 macrophages and 40.2 ± 4.7% control macrophages).

These results suggest that at least in our in vivo experimental conditions, there are no differences in the uptake of apoptotic cells between macrophages of diseased-BWF1 mice compared to age-matched controls mice.

### Peritoneal macrophages from diseased-BWF1 mice secrete higher levels of cytokines than control mice when activated with TLRs agonists

**Fig. 3:**
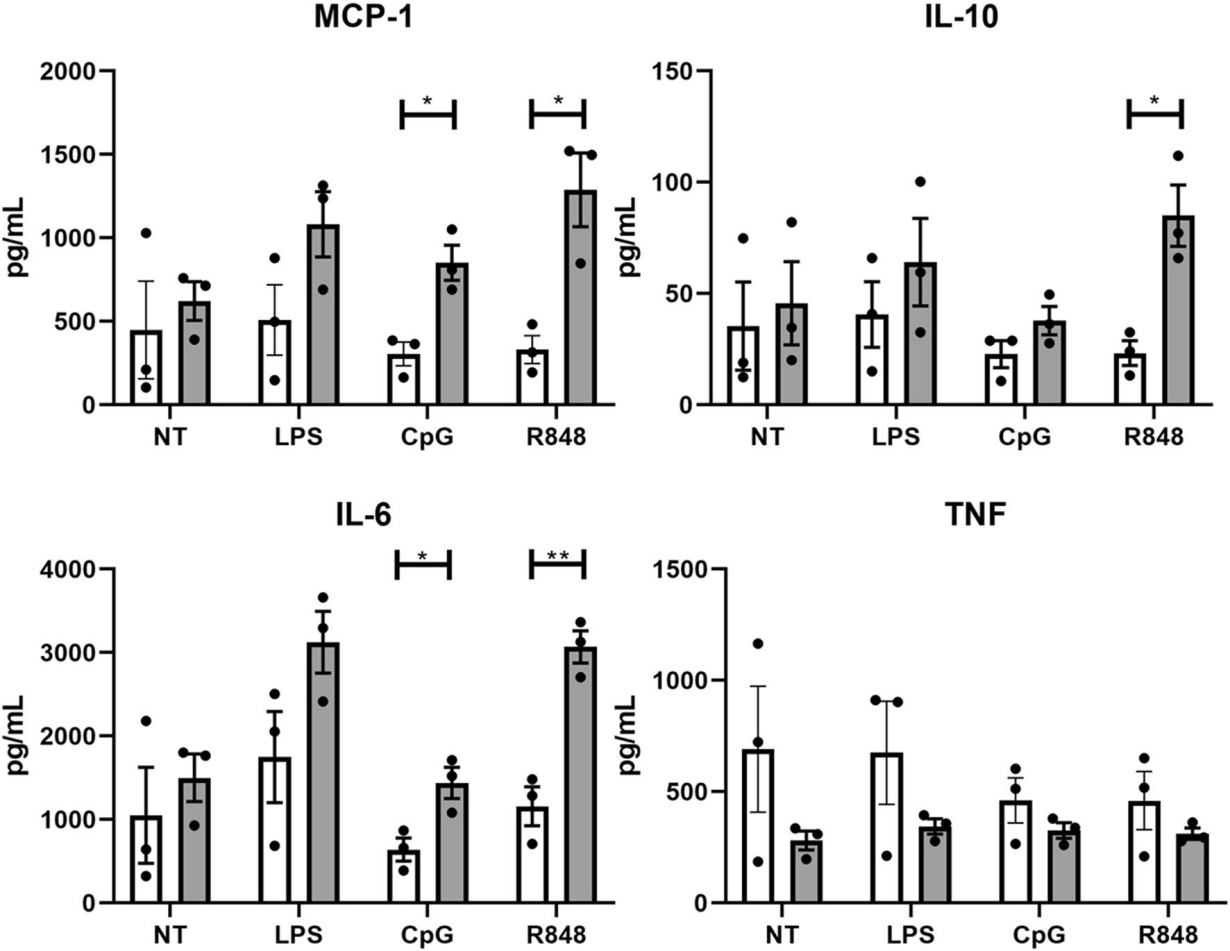
Cytokine production by peritoneal macrophages from diseased-BWF1 and control mice. Peritoneal macrophages from diseased-BWF1 (grey bars) or age-matched control mice (white bars) were cultured in the presence or absence (NT) of different stimuli (LPS, CpG, R848) for 20 h. Cytokine concentrations in the supernatant were determined using the CBA mouse inflammatory kit. Each bar represents the mean of 3 independent experiments. Student's t-test was used with *p < 0.05; **p < 0.01. (Color figure online)

Macrophages can produce a wide variety of cytokines depending on the microenvironment where they reside. Defects in cytokine secretion patterns may affect host defense and the development of inflammatory diseases [22, 23]. To elucidate the cytokine secretion profile of PMs, we sorted these cells from diseased-BWF1 and age-matched control mice and activated them for 20 h in the presence of LPS, R848, and CpG (TLR4, TLR7/8, and TLR9 agonists respectively). As shown in Fig. 3, PMs already produce a significant amount of TNF-a, MCP-1, IL-10, and IL-6 when cultured for 20 h in vitro without any stimulation, suggesting that they might be pre-activated in the peritoneal cavity. Additionally, we observed that PMs from diseased-BWF1 incubated with CpG produced a significantly higher amount of MCP-1 (monocyte chemoattractant protein-1) and IL-6 compared to control mice. When stimulated with R848, PMs from diseased-BWF1 produce more MCP-1, IL-6, and IL-10 compared to control mice. The concentration of IL-12 and IFN-g were under the detection limit. These results demonstrate that PMs from diseased mice secrete more pro-inflammatory cytokines than control mice when stimulated with intracellular TLRs agonists such as TLR7/9 that have been linked to SLE.

### Peritoneal macrophages suppress LPS-activated B cells

**Fig. 4:**
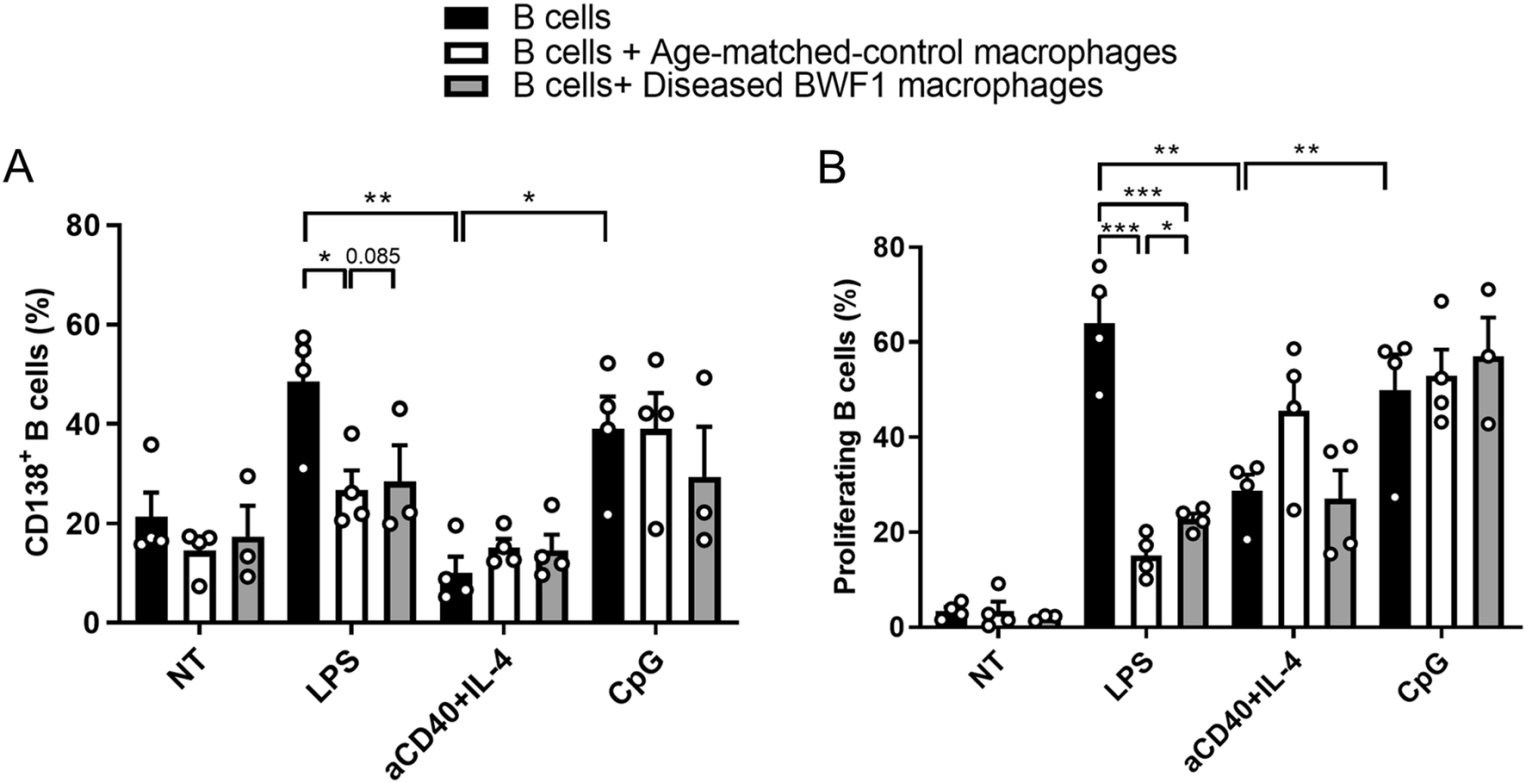
Peritoneal macrophages suppress B cell differentiation and proliferation induced by LPS. Peritoneal B cells from control mice were activated with different stimuli in the presence or absence of PMs from diseased-BWF1 or age-matched control mice. After 4 days, the differentiation of B cells into CD19 + /CD138 + pre-plasmatic cells (**A**) and their proliferation (**B**) was evaluated. Each bar represents the mean of 3 or 4 independent experiments. Student's t-test was used with *p < 0.05; **p < 0.01; ***p < 0.001

PMs and B cells are the most abundant cell populations in the peritoneal cavity. Since we observed phenotypic and functional differences in PMs obtained from BWF1 mice compared to control mice, we sought to determine whether PMs may affect peritoneal B cell proliferation and differentiation. For this, we cocultured total B cells with control or BWF1-derived PMs in the presence of different stimuli (usually used for B cell activation) to analyze the effect of PMs on B cell proliferation and differentiation into pre-plasmatic cells (CD138 +). We observed that LPS and CpG induce a higher differentiation and proliferation of B cells than untreated and αCD40 + IL-4 activated B cells (Fig. 4A and B). In all conditions, we observed that the addition of PMs to B cell cultures, reduced B cell proliferation and differentiation. Importantly, although macrophages from both, control and diseased mice significantly reduced LPS-induced B cell proliferation (Fig. 4B), PMs from BWF1-diseased mice were less efficient. On the other hand, only PMs from control mice reduced LPS-induced B cell differentiation (Fig. 4A). Furthermore, macrophages from both control and diseased mice do not affect B cell proliferation, or differentiation when B cells were stimulated with CpG or αCD40 + IL-4. We obtained similar results when we performed this experiment in transwell chambers, suggesting that PMs' effect on B lymphocytes is contact-independent (Supplementary Fig. 2). Since during an inflammatory process, both B cells and PMs will be exposed to different stimulus, we believe that our experimental strategy represents more physiological approach than only activating B cells.

### Peritoneal macrophages from diseased-BWF1 mice have a different transcriptional profile

**Fig. 5:**
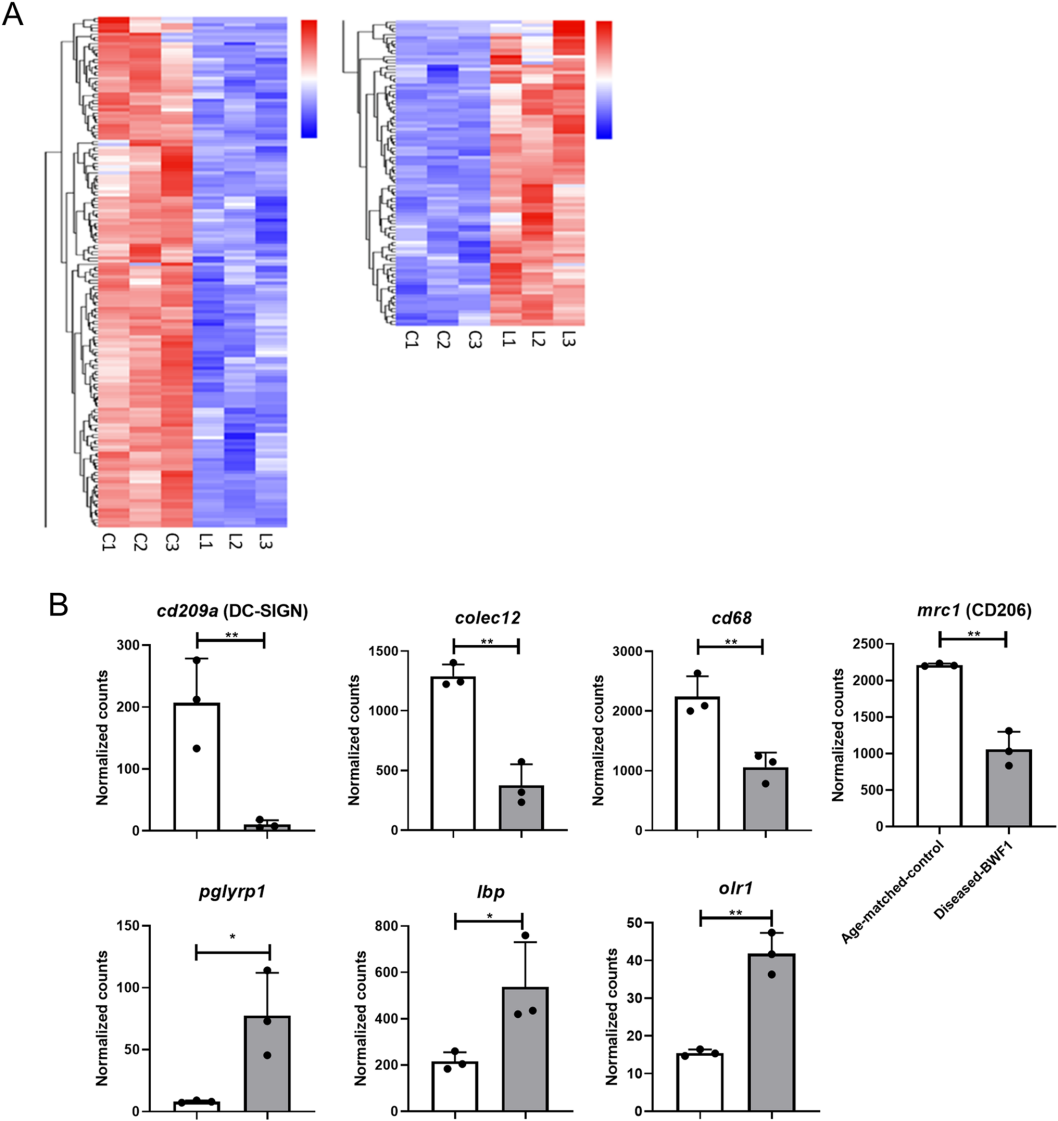
Peritoneal macrophages from diseased-BWF1 mice have a different transcriptional profile compared to age-matched control mice. RNAseq analysis of cell sorted PM (CD11b + F4/80 + CD19-) from diseased-BWF1 and age-matched control mice. **A** Heatmap shows genes downregulated (left) or upregulated (right) with 1.5-fold change and adjusted p value < 0.05 (list of genes in S1 and S2 table). Each column represents one mouse. **B** Normalized counts for selected molecules. White bars: age-matched control mice; gray bars: diseased-BWF1 mice. Each dot represents one mouse. Student's t-test was used with *p < 0.05; **p < 0.01. (Color figure online)

Next, we performed RNA sequencing (RNAseq) analysis to compare PM’s transcription profile in diseased-BWF1 and age matched-control mice. The subsequent statistical analysis led us to identify 106 genes upregulated and 180 genes downregulated in PMs from diseased-BWF1 mice compared to age-matched control mice (Fig. 5, Supplementary tables 1 and 2).

In our RNAseq analysis, we confirmed that CD206, an M2 marker, is downregulated in diseased mice compared to control mice, suggesting a bias from the M2 regulatory phenotype. However, commonly associated in vivo M1 markers such as CD86, CD38, G-protein coupled receptor 18 (*Gpr18*), formyl peptide receptor 2 (*Fpr2*), *STAT1*, and the M2-exclusive genes *Arg1*, early growth response protein 2 (*Egr2*), *STAT6*, and *c-Myc* [24] showed no difference between PMs from diseased or control mice.

On the other hand, macrophages can sense their environment through several receptors involved in recognizing pathogen-derived molecules, apoptotic cells, and soluble molecules capable of inducing specialized activation programs [22]. Our RNA-seq analysis showed differences in some of these receptors. We found CD209 (DC-SIGN), Colec12, CD68, and *Mrc1* (CD206) among the downregulated genes. In contrast, we found upregulation of the genes for a Peptidoglycan recognition protein 1 (*Pglyrp1*), lipopolysaccharide-binding protein (*Lbp*), Oxidized low-density lipoprotein receptor 1 (*Olr1*) in PMs from diseased mice compared to control mice (Fig. 5B). These data suggest that PMs from BWF1 mice present an altered transcriptional profile, which may have an impact on how they sense their microenvironment.

## Discussion

Macrophages are involved in many diseases, and they constitute an attractive therapeutic target since their plasticity allows the modification of their function to alter the disease outcome. However, it is necessary to determine and compare macrophage functions and phenotype in diseased and healthy individuals for these therapies to be effective. Several studies involved in the characterization of peritoneal macrophages (PMs) have been carried out with macrophages isolated after thioglycolate treatment, which are phenotypically and functionally different from resident macrophages [25, 26]. In this work, we focused on analyzing the phenotype and function of peritoneal cavity macrophages during homeostasis and the development of autoimmune disease in the BWF1 lupus-prone mice.

In this study, we found that the frequency of PMs decreases with age in both control and BWF1 mice, in agreement with previous findings [27]. However, the frequency and the absolute number of macrophages in 5-month-old and diseased-BWF1 mice are higher compared to age-matched-control mice. We speculate that specific signals in the peritoneal cavity of these mice might favor the proliferation and survival of peritoneal macrophages. Moreover, the higher frequency observed in peritoneal macrophages in 5-month-old BWF1 mice occurs before they present proteinuria and autoantibody production, suggesting that the alteration of macrophages precedes the onset of the disease. More experiments are needed to demonstrate the involvement of peritoneal macrophages in the development or onset of SLE.

On the other hand, in diseased-BWF1 mice, we observed a significantly higher frequency of the B1a subpopulation in the peritoneal cavity when compared with control mice. Peripheral blood B1a lymphocytes have been linked to autoimmune diseases such as autoimmune hemolytic anemia (AHA), Sjögren's syndrome, and autoimmune arthritis [28, 29]. Interestingly, Murakami's group reported that removing B1a lymphocytes from the peritoneal cavity in NZB and BWF1 mice reduced anti-DNA antibodies in serum and reduced pathological signs of SLE in the kidney [30].

Some reports indicate that B1a cells migrate towards BLC/CXCL13 mainly produced by peritoneal macrophages [19]. Thus, it is possible that the higher PM frequency in the diseased mice might explain the higher accumulation of B1a cells observed in these mice. However, our RNAseq analysis shows lower gene expression of CXCL13 in PM from BWF1 diseased mice compared to control mice (Supplementary Table 2). Thus, it would be interesting to analyze whether this correlates with the protein levels of CXCL13 chemokine and its receptor CXCR5 in PM and peritoneal B cell from diseased-BWF1 and control mice. In addition, it is possible that the accumulation of B1a lymphocytes allows the migration of B1a cells to other niches, such as the thymus, as shown in previous work from our group [31].

When analyzing the phenotype of PMs by flow cytometry, we observed a significant decrease in SIRPα, MHC-II, and CD206 expression compared to age-matched control mice. The expression of SIRPα is relevant since it binds to the CD47 molecule preventing the phagocytosis of healthy self-cells and tissue damage [32]. On the other hand, it has been reported that the mannose receptor CD206 is decreased in peritoneal and renal macrophages from B6.MRL-Fas^*lpr*^ mice compared to control mice. Additionally, the same group has demonstrated that macrophages in SLE patients express lower levels of CD206 compared to healthy controls [18]. The increase in the production of pro-inflammatory cytokines and the reduction of SIRPα and CD206 that we observed in BWF1 macrophages suggests that during autoimmune disease, the phenotype of PMs switches towards a more pro-inflammatory phenotype. Along with MHC-II reduction, it is also possible to speculate that PMs from diseased mice may have an altered antigen-presenting function, as demonstrated in SLE patients [33].

Macrophages polarize into classic (M1) pro-inflammatory or alternative (M2) anti-inflammatory macrophages to acquire specialized functional phenotypes in response to a combination of stimuli. In vitro*,* M1 and M2 macrophages show plasticity, while in vivo, there is a spectrum of phenotypes with M1 and M2 at the extremes [34]. Even though we observed by flow cytometry and RNAseq that the M2 marker CD206 was downregulated in diseased-BWF1 mice, we found by RNAseq that other common markers associated with M1 or M2 phenotype in vivo were not differentially expressed. Our result suggests that a broad spectrum of macrophages subpopulations might exist; however, in lupus-prone mice, macrophages present a more pro-inflammatory phenotype.

In addition to CD206, in our RNAseq analysis we found other upregulated and downregulated genes related to pathogen and scavenger receptors. We detected that *CD209*, *Colec12*, and *CD68* were downregulated in macrophages from diseased-BWF1 mice. The C-type lectin CD209 is involved in recognizing viruses and other pathogens, while Colec12 and CD68 are scavenger receptors that recognize non-self (mostly bacterial products) and altered (oxidized, acetylated) self-targets [35, 36]. On the other hand, *Pglyrp1*, *Lbp*, and *Olr1* are upregulated in diseased mice. In serum samples from SLE patients, it has been reported overexpression of *Pglyrp1*[37], a member of the family of bacterial peptidoglycan sensor molecules, and also it was associated with other autoimmune diseases like rheumatoid arthritis [38] Besides, SLE severity is associated with *Olr1* [4, 39]. The dysregulation of these sensor molecules may affect peritoneal homeostasis during disease and might contribute to susceptibility to infection in SLE.

Deficient phagocytosis by macrophages with the consequent accumulation of apoptotic cells and cellular debris has been suggested to contribute to the development of chronic inflammation and autoimmune disorders, including SLE [40, 41]. However, in our in vivo experiment, we observed no differences in the uptake of apoptotic cells between peritoneal macrophages from diseased BWF1 and age-matched control mice. In this line, a report has shown no differences in the phagocytic capacity of macrophages derived from blood monocytes in SLE patients, but rather a defect in serum soluble elements that might affect phagocytosis [42]. Also, it is important to consider that most studies showing altered phagocytosis by macrophages from lupus-prone mice and SLE patients are carried out in vitro*,* with bone-marrow derived macrophages or macrophages generated from circulating monocytes in the case of patients [41]. Thus, these conflicting results may be explained considering the differences in their experimental approach and models used.

Macrophages are the cytokine-producing cells by excellence. Therefore, we compared the ability of PMs from control and diseased mice to produce cytokines in response to different stimuli including TLR7 and TLR9 agonists that have been related to SLE [43–45]. We observed that TLR7 or TLR9 agonists induce higher amounts of MCP-1 and IL-6 on PMs from lupus-prone mice than control mice, confirming that PMs from diseased mice present a more pro-inflammatory phenotype. We also observed that only the TLR7 agonist induces the production of higher amounts of IL-10 in BWF1 mice. In line with our results, it has been reported that IL-6 and IL-10 are elevated in the blood of patients with SLE [46], while IL-6 has been associated with autoantibody production and disease development [47]. On the other hand, MCP-1, a chemoattractant of monocytes/macrophages, is used as a biomarker for lupus nephritis [48]. Given the role of these cytokines in the development of SLE, it is possible to postulate that deregulation in the production of these cytokines by peritoneal macrophages would contribute to the pathogenesis of the disease.

When analyzing the role of macrophages on B cell proliferation, we observed that PMs can suppress B cell proliferation, but PMs from diseased mice were less efficient than PM from control mice, again suggesting an altered function of PMs from BWF1 diseased mice. Riggs and collaborators [49] have shown that peritoneal macrophages suppress LPS-induced B cell proliferation through the production of prostaglandins and IL-10. Interestingly, as in our results, they also showed that macrophages did not reduce B cell proliferation when B cells were activated by CD40 ligation, suggesting that macrophage inhibition of B cell proliferation depends on the signaling pathways triggered on B cells. Moreover**,** the lower regulatory activity of peritoneal macrophages over B cell proliferation, together with higher levels of pro-inflammatory cytokines, may also explain, in part, the higher proportion of B1a cells in total B cells, observed in the peritoneal cavity in diseased mice.

Therefore, we speculate that the increase of macrophages with an altered phenotype and the accumulation of B1a cells in the peritoneal cavity of diseased BWF1 mice may increase anti-DNA antibodies in SLE, promoting the progression of the disease.

## Supplementary Information

Below is the link to the electronic supplementary material.

Supplementary file1 (DOCX 22 kb)

Supplementary file2 (DOCX 22 kb)

Supplementary file3 (TIF 495 kb) Peritoneal macrophages from diseased-BWF1 and age-matched control mice have the same engulfment capacity. Fluorescent-labeled apoptotic thymocytes (CellTrace Violet+) were adoptively transferred into diseased and control mice. After 45 minutes, the percentage of apoptotic cells that were uptaken by PMs was analyzed by FACS. (A) Representative plots depicting the percentage of thymocytes that have been internalized by macrophages, free thymocytes and thymocytes that bind to macrophages. Bar graph shows the frequency of apoptotic thymocytes that had been internalized. (B) Representative plot depicting the percentage of peritoneal macrophages that have internalized (CTV+) and not internalized apoptotic cells (CTV-). Bar graphs shows the frequency of PMs containing apoptotic thymocytes (cell trace violet+ in F4/80+ gate). Each bar represents the mean of 3 or 4 independent experiments

Supplementary file4 (TIF 285 kb) Peritoneal macrophages suppress B cell differentiation and proliferation induced by LPS in transwell chambers. Peritoneal B cells from control mice were activated with different stimuli in the presence or absence of PMs from diseased-BWF1 or age-matched control mice. After 4 days, the differentiation of B cells into pre-plasmatic cells (A) and their proliferation (B) was evaluated. Each bar represents the mean of 2 independent experiments (n=2)
