# Supplementary material for: Phenotypic and functional alterations of peritoneal macrophages in lupus-prone mice
Source: Mol Biol Rep. 2022 Feb 24;49(6):4193–204. doi: 10.1007/s11033-022-07252-0 (PMC9262788; doi:10.1007/s11033-022-07252-0)
Supplement: Supplementary file 1 — Supplementary file1 (DOCX 22 kb) [file 11033_2022_7252_MOESM1_ESM.docx]

**S1 Table 1. List of 106 upregulated genes in PM from diseased-BWF1 mice compared to control.** The genes in the list were selected with at least 1.5-fold change and p-value < 0.05.

| **MGI** | **log2FC** | **adj. P.Val** | **MGI** | **log2FC** | **adj. P.Val** | **MGI** | **log2FC** | **adj. P.Val** |
| --- | --- | --- | --- | --- | --- | --- | --- | --- |
| Cnbd2 | 4,120 | 1,E-74 | Orm1 | 5,982 | 2,E-03 | Appl2 | 0,874 | 2,E-02 |
| Cavin1 | 4,382 | 8,E-48 | Ahcy | 5,081 | 2,E-03 | Ikbke | 0,790 | 2,E-02 |
| Tmem154 | 3,998 | 1,E-23 | Ier2 | 0,747 | 2,E-03 | Phc3 | 0,675 | 2,E-02 |
| S1pr5 | 1,405 | 7,E-16 | F7 | 0,707 | 2,E-03 | Pogz | 5,871 | 3,E-02 |
| Cd52 | 3,460 | 8,E-16 | Coro1a | 2,409 | 2,E-03 | Ckap5 | 7,869 | 3,E-02 |
| Slc1a5 | 1,846 | 9,E-12 | Fmnl1 | 1,209 | 3,E-03 | Orm2 | 4,024 | 3,E-02 |
| Sell | 2,065 | 2,E-11 | Dyrk1a | 6,289 | 3,E-03 | Clec4e | 0,550 | 3,E-02 |
| Hp | 1,995 | 6,E-10 | Lbp | 1,316 | 3,E-03 | Prcp | 0,505 | 3,E-02 |
| Col22a1 | 8,954 | 4,E-09 | H13 | 1,015 | 3,E-03 | Chsy1 | 1,859 | 3,E-02 |
| Slc4a5 | 8,232 | 1,E-07 | Siglece | 1,684 | 4,E-03 | Arl14ep | 1,212 | 3,E-02 |
| Gpr132 | 8,141 | 2,E-07 | Gigyf2 | 1,138 | 4,E-03 | Rassf5 | 0,600 | 3,E-02 |
| Mmp14 | 3,221 | 6,E-07 | Rsad1 | 6,433 | 5,E-03 | Fblim1 | 1,270 | 3,E-02 |
| Tstd2 | 8,227 | 1,E-06 | Fosb | 7,058 | 5,E-03 | Arid3a | 0,931 | 3,E-02 |
| Pglyrp1 | 3,261 | 3,E-06 | Sla | 1,125 | 5,E-03 | Sh3pxd2a | 7,114 | 3,E-02 |
| Tmem176b | 1,206 | 4,E-06 | Rnf150 | 3,066 | 6,E-03 | Ntn4 | 1,996 | 4,E-02 |
| Junb | 0,854 | 8,E-06 | Tgm2 | 1,207 | 6,E-03 | Itgb5 | 3,626 | 4,E-02 |
| Leng8 | 0,625 | 3,E-05 | C3 | 1,222 | 7,E-03 | Rps6 | 1,032 | 4,E-02 |
| Sbno2 | 1,016 | 3,E-05 | Zfp420 | 6,195 | 7,E-03 | Enpp4 | 1,195 | 4,E-02 |
| Ids | 2,384 | 3,E-05 | Efnb2 | 0,882 | 8,E-03 | Rflnb | 3,197 | 4,E-02 |
| Sh3bgrl3 | 0,678 | 4,E-05 | Bace1 | 6,039 | 8,E-03 | Zfp595 | 2,089 | 4,E-02 |
| Rpl30 | 1,345 | 5,E-05 | Acvr1 | 1,011 | 8,E-03 | Casp8ap2 | 0,664 | 4,E-02 |
| Itga5 | 1,507 | 7,E-05 | Rps6ka1 | 0,740 | 8,E-03 | Ptprv | 2,687 | 4,E-02 |
| Rps6kb1 | 2,486 | 9,E-05 | Ptprs | 1,920 | 8,E-03 | P2rx1 | 1,471 | 4,E-02 |
| Ptges | 1,014 | 9,E-05 | Fos | 2,104 | 9,E-03 | Myo18a | 1,237 | 4,E-02 |
| Wipf1 | 0,676 | 1,E-04 | Rfx1 | 1,456 | 1,E-02 | Ccdc88b | 0,929 | 4,E-02 |
| Cd9 | 1,421 | 1,E-04 | Marveld1 | 0,785 | 1,E-02 | Smad5 | 0,731 | 4,E-02 |
| Chil1 | 8,676 | 2,E-04 | Nhlrc2 | 2,123 | 1,E-02 | Prr14l | 4,590 | 4,E-02 |
| Thbs1 | 2,877 | 2,E-04 | Htra3 | 0,714 | 1,E-02 | Tmem176a | 0,963 | 5,E-02 |
| Plxnd1 | 1,099 | 2,E-04 | Cdc14a | 1,147 | 1,E-02 | Clec5a | 2,300 | 5,E-02 |
| Hnrnpa2b1 | 0,871 | 3,E-04 | Bicra | 2,990 | 1,E-02 | Dlg3 | 1,867 | 5,E-02 |
| S100a6 | 1,688 | 3,E-04 | Arhgap26 | 7,507 | 2,E-02 | Lyl1 | 0,826 | 5,E-02 |
| Rnf217 | 2,253 | 6,E-04 | Rpl6 | 0,707 | 2,E-02 | Prok2 | 3,444 | 5,E-02 |
| Rasgrp2 | 6,164 | 7,E-04 | Ace | 1,956 | 2,E-02 | Gmip | 0,733 | 5,E-02 |
| Tspan32 | 1,562 | 8,E-04 | Nckap1 | 5,978 | 2,E-02 | Gtf3c4 | 0,596 | 5,E-02 |
| Mknk2 | 1,337 | 9,E-04 | Olr1 | 1,427 | 2,E-02 |  |  |  |
| Slc16a3 | 1,583 | 1,E-03 | Serpine1 | 3,730 | 2,E-02 |  |  |  |
