# Supplementary material for: Phenotypic and functional alterations of peritoneal macrophages in lupus-prone mice
Source: Mol Biol Rep. 2022 Feb 24;49(6):4193–204. doi: 10.1007/s11033-022-07252-0 (PMC9262788; doi:10.1007/s11033-022-07252-0)
Supplement: Supplementary file 2 — Supplementary file2 (DOCX 22 kb) [file 11033_2022_7252_MOESM2_ESM.docx]

**S2 Table 2. List of 180 Downregulated genes in PM from diseased-BWF1 mice compared to control.** The genes in the list were selected with at least 1.5-fold change and p-value < 0.05.

| **MGI** | **log2FC** | **adj. P.Val** | **MGI** | **log2FC** | **adj. P.Val** | **MGI** | **log2FC** | **adj. P.Val** |
| --- | --- | --- | --- | --- | --- | --- | --- | --- |
| Ppfibp1 | -1,961 | 1,E-27 | Cep290 | -6,384 | 3,E-04 | Serpinb8 | -0,616 | 1,E-02 |
| Cgnl1 | -3,825 | 1,E-27 | Tceanc2 | -2,291 | 3,E-04 | Bank1 | -0,710 | 1,E-02 |
| Zfp991 | -3,747 | 3,E-14 | Zscan25 | -6,711 | 3,E-04 | Ccl24 | -1,608 | 1,E-02 |
| Apoc2 | -4,346 | 7,E-14 | Rreb1 | -2,585 | 3,E-04 | Ap1b1 | -1,715 | 1,E-02 |
| Dpp7 | -1,793 | 1,E-12 | Tfe3 | -0,937 | 4,E-04 | Rab31 | -0,494 | 1,E-02 |
| Hal | -2,231 | 1,E-12 | Hexa | -0,743 | 5,E-04 | Calm2 | -0,667 | 1,E-02 |
| Epas1 | -1,815 | 3,E-12 | Rab11a | -0,605 | 5,E-04 | Glmp | -0,571 | 1,E-02 |
| Pmp22 | -0,752 | 4,E-12 | Renbp | -1,226 | 5,E-04 | Gja1 | -3,538 | 1,E-02 |
| Glb1 | -2,623 | 4,E-12 | Rtf2 | -0,623 | 6,E-04 | Wwp2 | -0,818 | 1,E-02 |
| Cd209a | -4,336 | 1,E-11 | Micu1 | -0,848 | 6,E-04 | Rab3d | -1,541 | 1,E-02 |
| Hpse | -0,984 | 4,E-10 | Cbr2 | -2,629 | 7,E-04 | Hist1h2bc | -2,146 | 1,E-02 |
| Fcgr2b | -2,226 | 4,E-10 | Nagk | -1,144 | 7,E-04 | Gla | -0,986 | 2,E-02 |
| Lgals1 | -2,275 | 3,E-09 | P2ry14 | -1,203 | 7,E-04 | Slc36a1 | -0,958 | 2,E-02 |
| Dnase2a | -1,120 | 1,E-08 | Ctsd | -0,941 | 1,E-03 | Ergic3 | -0,724 | 2,E-02 |
| Zfp984 | -1,868 | 4,E-08 | Vopp1 | -0,868 | 1,E-03 | Hebp1 | -0,974 | 2,E-02 |
| Uap1l1 | -1,453 | 6,E-08 | Atp6v0a1 | -0,880 | 1,E-03 | Mfsd1 | -0,655 | 2,E-02 |
| Ifi44l | -9,170 | 9,E-08 | Lgmn | -0,928 | 1,E-03 | Cd300ld3 | -0,713 | 2,E-02 |
| Atp6v1a | -0,668 | 9,E-08 | Igf1 | -1,787 | 1,E-03 | Sfrp1 | -1,941 | 2,E-02 |
| Lilr4b | -1,023 | 2,E-07 | Trf | -0,819 | 2,E-03 | Haus2 | -0,807 | 2,E-02 |
| Dynlt1c | -1,243 | 4,E-07 | Wfdc17 | -1,219 | 2,E-03 | Pyroxd2 | -0,717 | 2,E-02 |
| Anxa7 | -0,776 | 4,E-07 | Lancl1 | -1,069 | 2,E-03 | Fcna | -0,749 | 2,E-02 |
| Atp6v1b2 | -0,621 | 4,E-07 | Ivns1abp | -0,806 | 2,E-03 | Gnaq | -0,479 | 2,E-02 |
| Grn | -0,615 | 6,E-07 | Arg2 | -4,321 | 2,E-03 | Cdkn2b | -1,693 | 2,E-02 |
| Gm15922 | -0,907 | 1,E-06 | Gba | -1,095 | 3,E-03 | Icam2 | -0,573 | 2,E-02 |
| Pianp | -1,723 | 1,E-06 | Enpp1 | -3,928 | 3,E-03 | Acss1 | -1,034 | 2,E-02 |
| Rftn1 | -1,259 | 3,E-06 | Palld | -3,065 | 3,E-03 | Pilrb1 | -1,265 | 2,E-02 |
| Oxct1 | -0,944 | 5,E-06 | Cd63 | -1,525 | 3,E-03 | Rora | -1,001 | 2,E-02 |
| Mrc1 | -1,061 | 6,E-06 | P4ha2 | -1,157 | 3,E-03 | Wnt2 | -1,297 | 2,E-02 |
| Nxpe5 | -1,921 | 8,E-06 | Soat1 | -0,739 | 3,E-03 | Plekhm1 | -0,765 | 3,E-02 |
| Serpinb6a | -1,040 | 1,E-05 | Acin1 | -1,243 | 3,E-03 | Snx11 | -0,914 | 3,E-02 |
| Agtrap | -0,829 | 2,E-05 | Mmp27 | -3,349 | 3,E-03 | Mlph | -3,708 | 3,E-02 |
| Fabp3 | -2,687 | 2,E-05 | Ndufs5 | -5,325 | 3,E-03 | Fam129b | -0,641 | 3,E-02 |
| Clec4g | -7,781 | 2,E-05 | Washc5 | -0,687 | 3,E-03 | Pld3 | -0,829 | 3,E-02 |
| Hgd | -7,536 | 3,E-05 | Cndp2 | -0,651 | 3,E-03 | C2 | -2,718 | 3,E-02 |
| Ctsb | -0,661 | 3,E-05 | Camk1 | -0,513 | 4,E-03 | Tspo | -0,587 | 3,E-02 |
| Scn1b | -1,065 | 3,E-05 | Pus1 | -1,441 | 4,E-03 | Snx24 | -1,175 | 3,E-02 |
| Gnpda1 | -1,188 | 3,E-05 | Anxa5 | -0,857 | 4,E-03 | Anxa6 | -0,605 | 3,E-02 |
| Hpgd | -2,014 | 3,E-05 | Cstb | -1,249 | 4,E-03 | Ralgps2 | -6,235 | 3,E-02 |
| Cd209b | -2,618 | 4,E-05 | Rnaset2a | -0,737 | 4,E-03 | Neo1 | -2,439 | 3,E-02 |
| Colec12 | -1,781 | 5,E-05 | Blvrb | -0,572 | 5,E-03 | Vat1 | -0,634 | 3,E-02 |
| Emb | -4,095 | 9,E-05 | Hnrnph1 | -0,947 | 5,E-03 | Fam213b | -0,939 | 3,E-02 |
| Ifnar2 | -0,704 | 1,E-04 | Gyg | -0,984 | 6,E-03 | Itga7 | -6,301 | 3,E-02 |
| Atp6v0e2 | -6,963 | 1,E-04 | Prickle1 | -1,807 | 6,E-03 | Tgfbi | -1,027 | 3,E-02 |
| Sh3bgrl2 | -3,381 | 1,E-04 | Cxcl13 | -1,459 | 6,E-03 | Plod1 | -0,644 | 4,E-02 |
| Itm2b | -0,475 | 1,E-04 | Rin2 | -0,667 | 6,E-03 | Hpn | -1,671 | 4,E-02 |
| Prdx1 | -0,641 | 1,E-04 | Lamp1 | -0,522 | 6,E-03 | Ppm1f | -8,193 | 4,E-02 |
| Galc | -1,117 | 1,E-04 | Entpd1 | -0,595 | 7,E-03 | P2rx4 | -0,569 | 4,E-02 |
| Naglu | -0,832 | 1,E-04 | Naga | -0,756 | 7,E-03 | Por | -0,590 | 4,E-02 |
| C1qa | -0,859 | 1,E-04 | Bloc1s3 | -7,156 | 7,E-03 | Ipo11 | -1,113 | 4,E-02 |
| Aldh2 | -0,639 | 1,E-04 | Amdhd2 | -1,354 | 7,E-03 | Pf4 | -1,143 | 4,E-02 |
| Abcd4 | -1,332 | 1,E-04 | C130026I21Rik | -1,119 | 8,E-03 | Tnks1bp1 | -8,142 | 4,E-02 |
| Gfra2 | -4,129 | 1,E-04 | Cdk8 | -1,093 | 8,E-03 | Wdr41 | -0,591 | 4,E-02 |
| Ctsa | -0,935 | 2,E-04 | Cln5 | -0,616 | 8,E-03 | Selenow | -0,573 | 4,E-02 |
| Prr13 | -0,523 | 2,E-04 | AW554918 | -6,692 | 8,E-03 | Mcu | -0,674 | 4,E-02 |
| C1qc | -0,777 | 2,E-04 | Prnp | -0,520 | 8,E-03 | Col23a1 | -1,474 | 4,E-02 |
| Adk | -0,933 | 2,E-04 | Ehbp1 | -6,120 | 1,E-02 | Tlr7 | -0,916 | 4,E-02 |
| Crtap | -1,012 | 2,E-04 | Fam19a5 | -6,341 | 1,E-02 | Cln8 | -0,622 | 5,E-02 |
| Selenop | -1,348 | 2,E-04 | Pnpla7 | -0,805 | 1,E-02 | Mdm1 | -0,869 | 5,E-02 |
| Cd68 | -1,080 | 3,E-04 | Blnk | -1,301 | 1,E-02 | Car13 | -1,776 | 5,E-02 |
| Galnt9 | -5,610 | 3,E-04 | Tpp1 | -0,556 | 1,E-02 | Hip1 | -1,810 | 5,E-02 |
