# Supplementary figures and images for: Phenotypic and functional alterations of peritoneal macrophages in lupus-prone mice
Source: Mol Biol Rep. 2022 Feb 24;49(6):4193–204. doi: 10.1007/s11033-022-07252-0 (PMC9262788; doi:10.1007/s11033-022-07252-0)

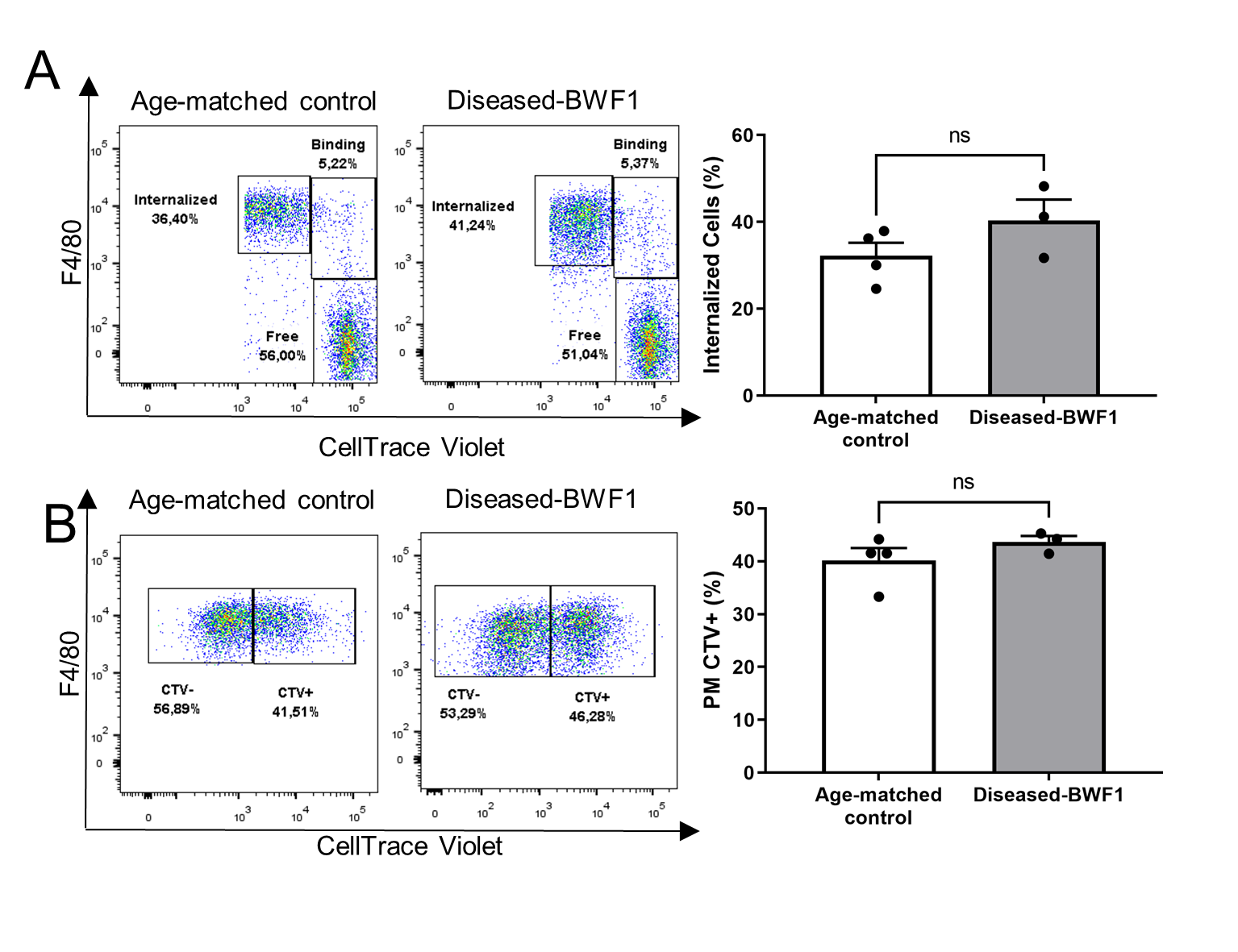

Supplement: Supplementary file 3 — Supplementary file3 (TIF 495 kb) Peritoneal macrophages from diseased-BWF1 and age-matched control mice have the same engulfment capacity. Fluorescent-labeled apoptotic thymocytes (CellTrace Violet+) were adoptively transferred into diseased and control mice. After 45 minutes, the percentage of apoptotic cells that were uptaken by PMs was analyzed by FACS. (A) Representative plots depicting the percentage of thymocytes that have been internalized by macrophages, free thymocytes and thymocytes that bind to macrophages. Bar graph shows the frequency of apoptotic thymocytes that had been internalized. (B) Representative plot depicting the percentage of peritoneal macrophages that have internalized (CTV+) and not internalized apoptotic cells (CTV-). Bar graphs shows the frequency of PMs containing apoptotic thymocytes (cell trace violet+ in F4/80+ gate). Each bar represents the mean of 3 or 4 independent experiments [file 11033_2022_7252_MOESM3_ESM.tif]

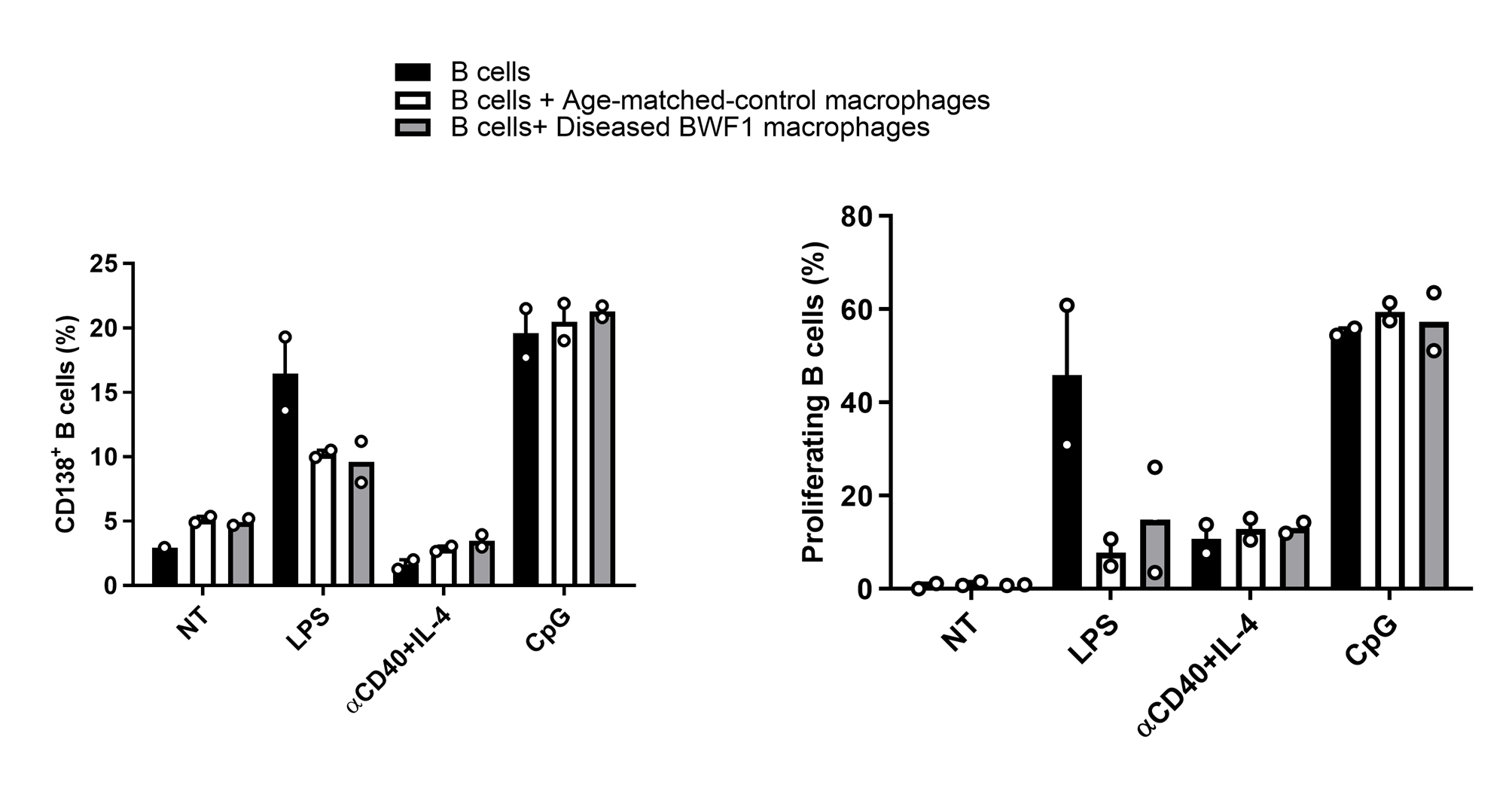

Supplement: Supplementary file 4 — Supplementary file4 (TIF 285 kb) Peritoneal macrophages suppress B cell differentiation and proliferation induced by LPS in transwell chambers. Peritoneal B cells from control mice were activated with different stimuli in the presence or absence of PMs from diseased-BWF1 or age-matched control mice. After 4 days, the differentiation of B cells into pre-plasmatic cells (A) and their proliferation (B) was evaluated. Each bar represents the mean of 2 independent experiments (n=2) [file 11033_2022_7252_MOESM4_ESM.tif]
